# *ARMC5* mutations are associated with high levels of proliferating cell nuclear antigen and the presence of the serotonin receptor 5HT4R in PMAH nodules

**DOI:** 10.20945/2359-3997000000236

**Published:** 2020-03-30

**Authors:** Barbara Brito da Conceição, Isadora Pontes Cavalcante, Jean Lucas Kremer, Thais Barabba Auricino, Eduarda Corrêa Bento, Maria Claudia Nogueira Zerbini, Maria Candida Barisson Villares Fragoso, Claudimara Ferini Pacicco Lotfi

**Affiliations:** 1 Instituto de Ciências Biomédicas Departamento de Anatomia Universidade de São Paulo São Paulo SP Brasil Instituto de Ciências Biomédicas, Departamento de Anatomia, Universidade de São Paulo, São Paulo, SP, Brasil; 2 Departamento de Patologia Universidade de São Paulo São Paulo SP Brasil Departamento de Patologia, Universidade de São Paulo, São Paulo, SP, Brasil; 3 Laboratório de Hormônios e Genética Molecular LIM/42 Universidade de São Paulo São Paulo SP Brasil Laboratório de Hormônios e Genética Molecular LIM/42, Universidade de São Paulo, São Paulo, SP, Brasil

**Keywords:** Cushing’s syndrome, adrenal hyperplasia, ARMC5, steroidogenic enzymes, aberrant G protein-coupled receptors, ectopic ACTH

## Abstract

**Objective:**

To analyze the morphological and functional characteristics of primary macronodular adrenal hyperplasia (PMAH) nodules carrying or not carrying ARMC5 mutations and the consequences of the presence of mutations in terms of the pattern of macronodule composition and functional state.

**Subjects and methods:**

The analyses were performed by hematoxylin-eosin staining, immunohistochemistry, microdissection of spongiocyte tissue and RT-qPCR of histological sections from 16 patients diagnosed with PMAH with germline (5) or germline/somatic mutations (5) and without mutations (6) in the *ARMC5* gene.

**Results:**

Hyperplastic nodules were predominantly composed of spongiocytes in mutated and nonmutated sections. ARMC5 mRNA expression in spongiocytes was higher in ARMC5-mutated nodules than in ARMC5-nonmutated nodules, and homogenous ARMC5 protein distribution was observed. The presence of arginine-vasopressin receptor (AVP1AR) and ectopic ACTH production were observed in both cell populations regardless of ARMC5 mutations; the numbers of serotonin receptor (5HT4R)- and proliferating cell nuclear antigen (PCNA)-positive cells were higher in macronodules carrying ARMC5 mutations than in those without mutations.

**Conclusions:**

Our results suggest that the presence of ARMC5 mutations does not interfere with the pattern of distribution of spongiocytes and compact cells or with the presence of AVP1AR, gastric-inhibitory polypeptide receptor (GIPR) and ectopic ACTH. Nevertheless, the higher numbers of PCNA-positive cells in mutated nodules than in nonmutated nodules suggest that mutated ARMC5 can be related to higher proliferation rates in these cells. In conclusion, our results provide more information about the crosstalk among abnormal GPCRs, ectopic ACTH in steroidogenesis and the *ARMC5* gene, which may be relevant in understanding the pathogenesis and diagnosis of patients with PMAH.

## INTRODUCTION

Primary macronodular adrenal hyperplasia (PMAH) is a rare cause of adrenal hypercortisolism, accounting for less than 2% of cases of Cushing’s syndrome (CS) (1). The cellular pattern of the macronodules consists of spongiocytes and compact cells that present with a differential presence of steroidogenic enzymes (2). However, it is not yet clear whether these cells are two different cell types or the same cell type in different functional states. PMAH nodules are generally characterized by the presence of aberrant receptors, including vasopressin (AVP), serotonin (5HT4) and gastric inhibitory peptide (GIP) receptors. In addition, ectopic ACTH production in clusters of PMAH cells is hypothesized to abnormally stimulate steroidogenesis by stimulating abnormal G protein-coupled receptors in the adrenal macronodules *in vitro* and *in vivo* (3-6).

As for the molecular cause of PMAH, mutations in the Armadillo repeat-containing 5 (*ARMC5*) gene were described in different cohorts of potentially sporadic and familial cases of PMAH (7-11). *ARMC5* is considered a putative tumor suppressor gene and an important regulator of cell apoptosis, steroidogenesis, development and the immune system in mice (7,11-14). However, the consequences of mutations in *ARMC5* for the development of PMAH remain to be established.

The aim of this study was to analyze the morphological and functional characteristics of PMAH nodules with and without ARMC5 mutations and whether mutations in the *ARMC5* gene interfere with the pattern of macronodule composition and the functional state of these cells.

## SUBJECTS AND METHODS

### Patient groups

For this study, we selected 16 nonrelated patients diagnosed with PMAH (Table 1). The sections of PMAH macronodule samples originated from 13 females and 3 males who underwent surgical procedures to treat hypercortisolism due to PMAH and were classified into 2 groups. **Group 1** consisted of 6 patients (5 females and 1 male) without mutations in the *ARMC5* gene, with a mean age of 57.0 ± 6.8 years, an average baseline urinary cortisol of 14.7 ± 6.3 µg/dL and a range of plasmatic ACTH between < 2 pg/mL and 16.9 pg/mL; one of these patients was diagnosed with overt Cushing’s syndrome (CS), and five patients were diagnosed with subclinical hypercortisolism. **Group 2 **consisted of 10 patients (8 females and 2 males) with *ARMC5* germline or germline and somatic mutations, a mean age of 51.1 ± 8.2 years, an average baseline urinary cortisol of 22.3 ± 6.3 µg/dL and an ACTH of < 5 pg/mL and CS diagnosis. The diagnosis of overt Cushing’s syndrome was defined by autonomous cortisol secretion after the dexamethasone suppression test (DST) and/or an increased 24-hour urinary cortisol and/or suppressed ACTH levels accompanied by clinical signs such as proximal myopathy, skin fragility and facial plethora. Subclinical hypercortisolism was established when the patient presented abnormal cortisol levels with autonomous serum cortisol secretion after DST without the clinical signs of Cushing’s syndrome. The clinical data from all patients are shown in Table 2. This study was approved by the Ethics Committee of the Institute of Biomedical Sciences of the University of São Paulo (nº 1288/CEPSH). Written informed consent was obtained from patients assisted at the Adrenal Unit of Service of Endocrinology and Metabolism of USP.

**Table 1:** *ARMC5* mutations identified in blood samples and adrenal tissue from the patients diagnosed with PMAH

Patient	Age	Gender	Group	Type of Mutation	

				Blood	Adrenal Tissue
**1**	63	F	G	c.476 -1G>C (het)	c.476 -1G>C (het)
**2**	56	F	G	c.1158G>A; p.Trp386* (het)	c.1158G>A; p.Trp386* (het)
**3**	52	M	G	c.280_281delTC, p.Ser94Valfs*8 (het)	c.280_281delTC, p.Ser94Valfs*8 (het)
**4**	41	F	G	c.170_171insA, I58Nfs*45 (het)	c.170_171insA, I58Nfs*45 (het)
**5**	47	F	G	c.799C>T, p.Arg267*, rs369721476 (het)	c.799C>T, p.Arg267*, rs369721476 (het)
**6**	49	F	G S	c.1181T>C, p.Leu394Prol (het)	c.1181T>C, p.Leu394Prol (het) c.1559_1559delG; Gly520Aspfs*24 (het)
**7**	45	F	G S	c.170_171insG, Ile58Asnfs*45 (het)	c.170_171insG, Ile58Asnfs*45 (het) Loss of heterozygosity (LOH)
**8**	45	M	G S	c.2423A>C, p.His808Pro (het)	c.2423A>C, p.His808Pro (het) c.283_295delTCGGCCGCGTCGGG,p.Ser95Serfs*2 (het)
**9**	45	F	G S	c.165_166insG, p.Ile58Asnfs*45, (het)	c.165_166insG, p.Ile58Asnfs*45, (het) c.2082_2088delCCCGCTC,p.Pro695Serfs*20, (het)
**10**	68	F	G S	c.1960C>T, p.Arg654*, (het)	c.1960C>T, p.Arg654*, (het) c.294_294delG; p.Gly99Glufs*38 (het)
**11**	53	F	NM		
**12**	50	F	NM		
**13**	50	M	NM		
**14**	69	F	NM		
**15**	61	F	NM		
**16**	59	F	NM		

PMAH: primary macronodular adrenal hyperplasia; G: germline; S: somatic; het: hetero; NM: non-mutated.

**Table 2:** Clinical data from patients diagnosed with PMAH

Patient	Cortisol (µg/dL)	ACTH (pg/mL)	Cortisol post DST (pg/mL)	Cushing/SH
**1**	25.9	<5	32.8	Cushing
**2**	29.9	<5	25.3	Cushing
**3**	20.4	<5	8.3	Cushing
**4**	16.3	16.9	4.9	SH
**5**	11.4	6.4	3.0	SH
**6**	19.9	<5	27.1	Cushing
**7**	21.8	<5	18.7	Cushing
**8**	26.6	<5	1.9	Cushing
**9**	11.2	<5	7.5	Cushing
**10**	30.5	<5	27.4	Cushing
**11**	8.7	<5	6.2	SH
**12**	25.7	<5	6.4	Cushing
**13**	27.7	<5	33.2	Cushing
**14**	10.7	3.2	2.7	SH
**15**	13.5	<2	13.9	SH
**16**	13.5	<2	13.9	SH

PMAH: primary macronodular adrenal hyperplasia; DST: dexamethasone suppression test; SH: subclinical hypercortisolism.

Basal cortisol reference value: 3,4-16,8 µg/dL. Cortisol reference value after DST values: < 1,8 µg/dL

### Analysis of cell type of the tissue sections from PMAH macronodules

The spongiocytes and compact cells of hyperplastic nodules were quantified in sections of PMAH nodules with or without germline and/or somatic mutations in *ARMC5*. The sections were stained with hematoxylin and eosin (HE) (Merck, Darmstadt, Germany) and were analyzed with a Nikon light microscope (MBF Bioscience, Williston, USA) by using Neurolucida software. Histological sections were submitted to a deparaffinization and rehydration protocol followed by staining with HE. The total area of the sections was delineated, and the area of compact cells was randomly analyzed in relation to the total area. The spongiocytes were quantified by subtracting the compact area from the total section area.

### Immunohistochemistry

Sections (7 µm) of formalin-fixed paraffin-embedded adrenal nodule tissue were obtained for immunohistochemistry reactions. For antigenic recovery, immersion in citric acid (pH 6.0) or Tris-EDTA buffer (pH 9.0) was used for 30 minutes at 96 °C. For blockade of endogenous peroxidase, methanol and H_2_O_2_ (1:1) were used for 20 minutes, followed by blockade of nonspecific sites with phosphate-buffered saline containing 0.1% Tween 20 (PBST) and 5% horse serum (ABC Vectastain Kit, Vector Laboratory, Burlingame, CA, USA) for 1 hour at room temperature (RT). After blockade, the sections were incubated overnight at 4°C with a primary antibody from Abcam (Cambridge Science Park, Cambridge, UK) or Santa Cruz (Santa Cruz Biotechnology, Santa Cruz, CA, USA) at the following concentrations: anti-StAR (1:200), anti-3βHSD2 (1:250), anti-PCNA (1:100), anti-ARMC5 (1:150), anti-ACTH (1:50), anti-CYP17A1 (1:100), anti-AVP1AR (1:50), anti-GIPR (1:1,000), and anti-5HT4R (1:50) diluted in PBST containing 5% serum in a humid chamber. Next, the sections were incubated with a universal biotinylated secondary antibody for 1 hour at RT, followed by incubation with the ABC Vectastain Kit AB (1:100) complex for 1 hour at RT. Finally, the sections were incubated with 3,3’-diaminobenzidine (DAB) (Sigma, St. Louis, MO, USA) and counterstained with hematoxylin.

### Immunostaining quantification

The images were captured with a light field microscope with a 20x and a 40x objective lens (Nikon, Eclipse 80i). Five images of each patient were randomly selected and analyzed by ImageJ 1.43j software (National Institutes of Health, Bethesda, Maryland, USA). By using the hematoxylin and DAB (HDAB) built-in vector, the images were convoluted in three parts as follows: hematoxylin, DAB and background. Only the DAB-stained images were selected and, when submitted to the threshold filter, converted into a binary image. The software calculated the percentage of area stained by DAB using the same parameters for each image.

### Analysis of gene expression

Frozen sections of PMAH tissue fragments were mounted on slides suitable for the ArcturusXT™ laser microdissection system (LCM) (ThermoFisher, Arcturus^®^, LCM0522). This technique was performed on a Nikon Eclipse^®^ Ti-E inverted microscope with a computerized Arcturus^®^ AutoScanXT™ system, which allows for the identification of areas to be microdissected. After locating the cells of interest, a CapSure^®^ LCM Cap was placed over the target area. The laser pulsed through the cap forming a thin protrusion that bridged the gap between the cap and tissue and adhered to the target cell. Lifting the cap transferred the target cells attached to the cap to a tube containing an RNA extraction buffer previously heated to 42 °C. The obtained material was centrifuged for 2 minutes at 12,300 rpm and stored at -80°C. Total RNA was extracted using the PicoPure^®^ RNA Isolation Kit, and cDNA was synthesized with SuperScript III First Strand Synthesis Supermix (Invitrogen, USA). The cDNA obtained was amplified by the SYBR Green method, and real-time PCR (qPCR) was used to analyze the relative gene expression levels of *ARMC5*,* 3BHSD2*,* CYP17A1*,* CYP11B1*,* 5HT4R*, *GIPR*, and *AVPRV1a*. The *GUSB* gene was used as an endogenous control, and the relative expression was analyzed using the 2^-^ method, where ΔCT is the difference between CT values selected from a given sample, and CT is from a commercial pool of human normal adrenal tissues (Clontech, Palo Alto, CA). The primers used in the qPCR experiments are described in Table 3.

**Table 3:** qPCR SybrGreen primers sequences

Gene	*Forward*	*Reverse*
***GUSB***	AGCCAGTTCCTCATCAATGG	GGTAGTGGCTGGTACGGAAA
***ARMC5***	CTCGGAGGCATACTCCCTTT	GTTCGGTTCTGGATGCTGTC
***3BHSD2***	GAGGCAGTAAGGACTTGGACT	CGTGGCCAATCCAAAGTAGC
***CYP17A1***	AGCCGCACACCAACTATCAGTGAC	TCACCGATGCTGGAGTCAACGTTG
***AVP V1a***	CGGCTTCATCTGCTACAACATC	CGAGTCCTTCCACATACCCGT
***5HT4***	CGGGCAGGAGCCTCCTCCGAGAG	CAAGGGACAGTCTGGCCCAGAATG
***GIPR***	CCTGATCGCCCCTGCACGAAC	AGGTCGAGGTAGCAGACGGTCTCG

### Statistical analysis

Data are presented as the mean ± standard deviation (SD). Statistical significance was determined using a paired Student’s t-test, and for more than two groups, ANOVA was used. The results were considered statistically significant when p < 0.05.

## RESULTS

**Figure 1:**
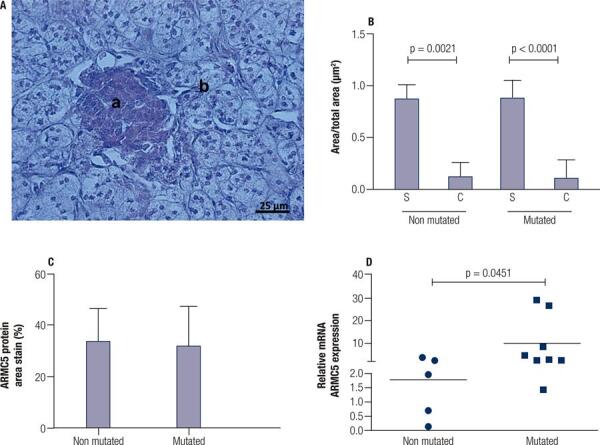
A) Representative section from a PMAH patient stained with hematoxylin and eosin, showing (a) compact cells and (b) spongiocytes. B) Area measurement containing spongiocytes (S) and compact cells (C) in sections containing hyperplasia from PMAH patients without and with ARMC5 gene mutations. C) Percentage of the area stained by ARMC5-positive cells from immunohistochemistry sections. D) Relative ARMC5 gene expression in spongiocytes obtained by microdissection from hyperplastic areas of tissues from PMAH patients without and with ARMC5 gene mutations. P-values were calculated using Student’s t-test.

**Figure S1:**
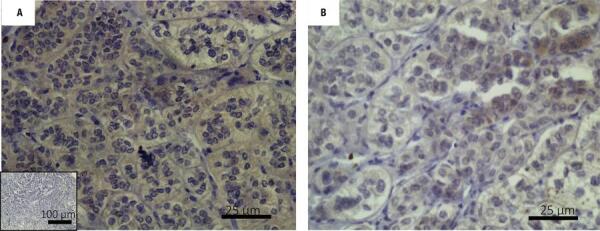
Immunohistochemical analysis of the ARMC5 protein showed similar quantities in the cytoplasm of both spongiocytes and compact cells in nonmutated (A) and mutated (B) PMAH patient tissues. Hyperplastic sections incubated in nonimmune primary sera yielded negative results (inset).

**Figure 2:**
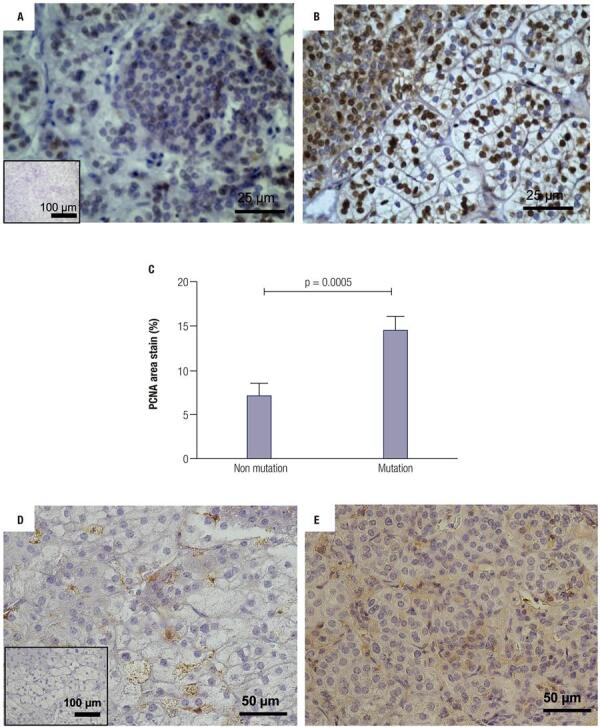
Immunohistochemical analysis of PCNA (A and B) and ARMC5 (D and E) proteins in hyperplastic sections of tissues from PMAH patients without (A and D) and with (B and E) ARMC5 gene mutations. Negative controls were generated by omitting the PCNA and ARMC5 antibodies (insets). C) Percentage of the area stained by PCNA-positive cells from immunohistochemistry sections. P-values were calculated using Student’s t-test.

**Figure S2:**
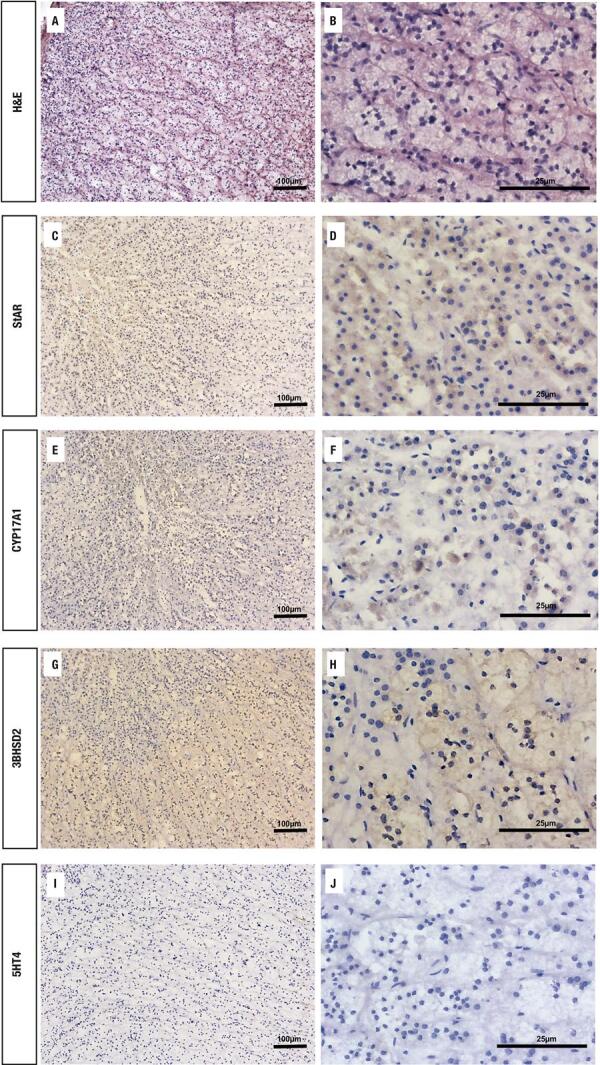
Representative sections from normal human adrenal tissues stained with hematoxylin and eosin (A) and (B). Immunohistochemical analysis of the StAR (C and D), CYP17A1 (E and F), 3BHSD2 (G and H) and serotonin receptor (5HT4R) proteins (I and J) in sections from normal human adrenal tissues.

**Figure 3:**
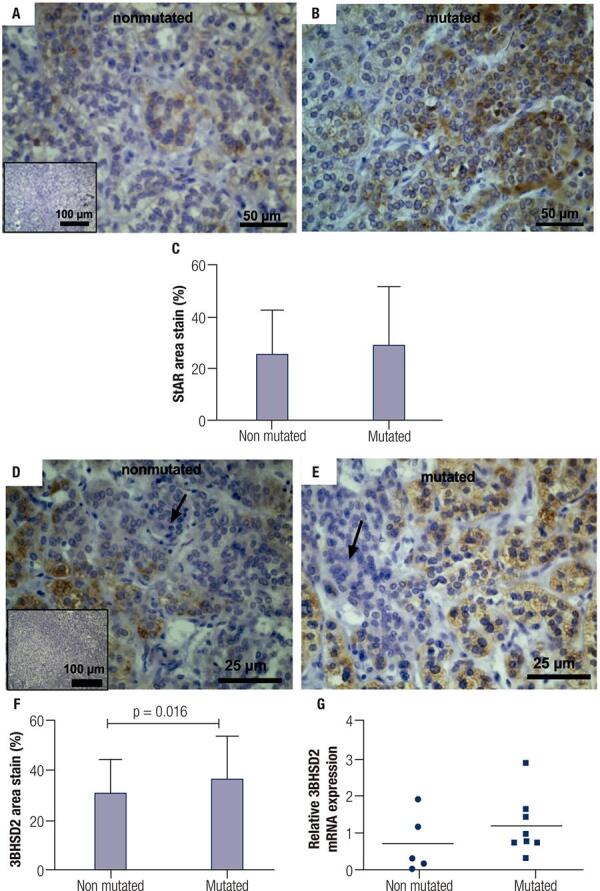
Immunohistochemical analysis of the StAR (A and B) and 3BHSD2 (D and E) proteins in hyperplastic sections in tissues from PMAH patients without (A and D) and with (B and E) ARMC5 gene mutations. The black arrow shows the 3BHSD2-negative compact cells. Negative controls were generated by omitting the PCNA and 3BHSD2 antibodies (insets). C) Percentage of the area stained by StAR-positive cells from immunohistochemistry sections. F) Percentage of the area stained by 3BHSD2-positive cells from immunohistochemistry sections. G) Relative 3BHSD2 gene expression in spongiocytes obtained by microdissection of hyperplastic tissues of PMAH patients without and with ARMC5 gene mutations. P-values were calculated using Student’s t-test.

**Figure S3:**
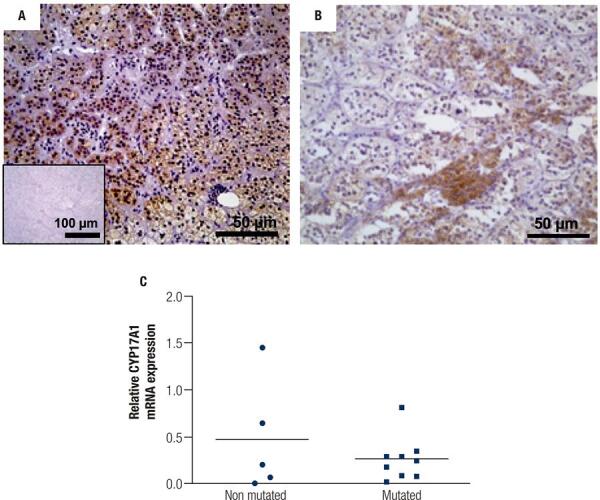
Immunohistochemical analysis of the CYP17A1 protein in sections of hyperplastic tissues from PMAH patients without (A) and with (B) ARMC5 gene mutations. Negative controls were generated by omitting the CYP17A1 antibody (inset). C) Relative CYP17A1 gene expression in spongiocytes obtained by microdissection of hyperplastic tissues from PMAH patients without and with ARMC5 gene mutations. P-values were calculated using Student’s t-test.

**Figure 4:**
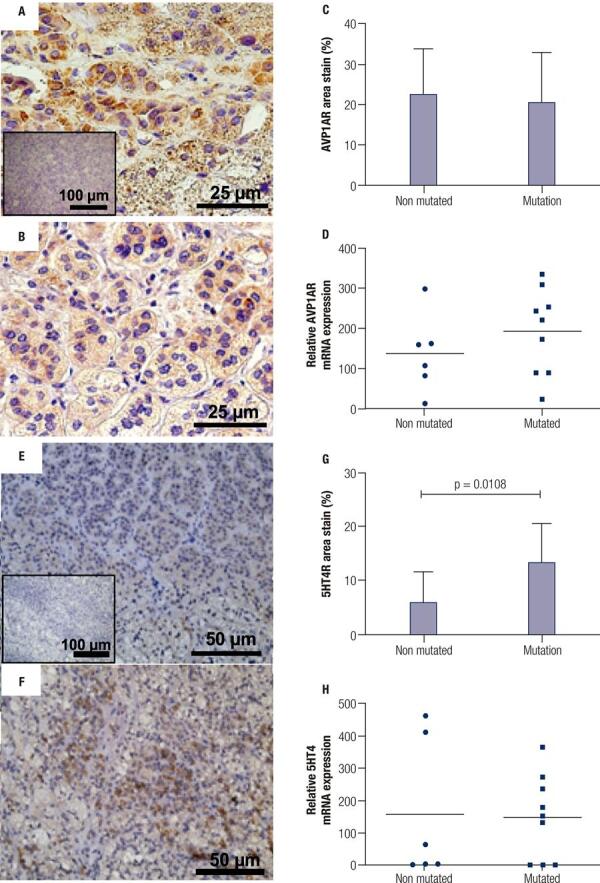
Immunohistochemistry of the arginine-vasopressin receptor (AVP1AR) and serotonin receptor (5HT4R) proteins in sections of hyperplastic tissues from PMAH patients without (A and E) and with (B and F) ARMC5 gene mutations. Negative controls were generated by omitting the AVP1AR and 5HT4R antibodies (insets). C) Percentage of the area stained by AVP1AR-positive cells and G) by 5HT4R-positive cells from immunohistochemistry sections. D) Relative AVP1AR gene expression and H) 5HT4R gene expression in spongiocytes obtained by microdissection of hyperplastic tissues from PMAH patients without and with ARMC5 gene mutations. P-values were calculated using Student’s t-test.

**Figure S4:**
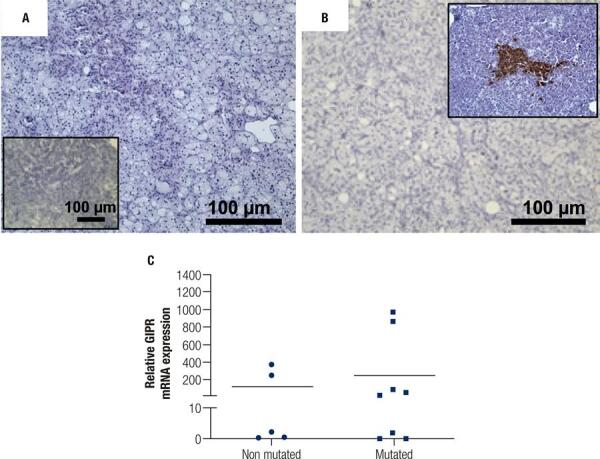
Immunohistochemistry of the gastric-inhibitory polypeptide receptor (GIPR) protein in sections from hyperplastic tissues from PMAH patients without (A) and with (B) ARMC5 gene mutations. Negative controls were generated by omitting the GIPR antibody (inset in A), and alpha-cells from pancreatic islets served as positive controls (inset in B). C) Relative GIPR gene expression in spongiocytes obtained by microdissection of hyperplastic tissues from PMAH patients with and without ARMC5 gene mutation. P-values were calculated using Student’s t-test.

To better characterize the tissue sections from patients who would be further analyzed, we investigated the cell types that constituted the PMAH nodules through histochemistry and immunostaining, and we determined the protein and gene expression levels of *ARMC5* in the mutated and nonmutated PMAH patient tissues (Figure 1). We observed that the nodules were composed predominantly of spongiocytes (Figures 1A and 1B) and that the ARMC5 protein was present in similar quantities in the cytoplasm of both spongiocytes and compact cell types in both mutated and nonmutated PMAH patient tissues (Figure S1; Figure 1C). For comparison, we performed hematoxylin and eosin staining in normal human adrenal tissue, as observed in Figures S2A and S2B. To perform an accurate analysis of the nodules of patients diagnosed with PMAH and given that spongiocytes were the predominant population in the nodules, we microdissected the areas containing spongiocytes and investigated gene expression related to markers previously analyzed by immunohistochemistry. We also observed that spongiocytes in sections from mutated PMAH patients presented higher expression of *ARMC5* mRNA than those in sections from nonmutated PMAH patients (Figure 1D). To investigate the proliferative potential of the cells, the S phase-related protein proliferating cell nuclear antigen (PCNA) was analyzed (15). We observed that both spongiocytes and compact cells presented similar patterns of PCNA protein distribution (Figures 2A and 2B). Moreover, patients carrying mutations in *ARMC5* showed more PCNA-positive cells than those not carrying mutations (p = 0.0005), with percentages of 14.7 1.3% and 7.2 ± 1.3%, respectively (Figure 2C). Additionally, we tested whether compact cells and spongiocytes could be responsible for different functions of the hyperplastic cells by investigating the expression of different proteins related to steroidogenesis in PMAH. First, we analyzed the production of ectopic ACTH in PMAH cells with a method previously described by Louiset and cols. (6). We observed that ectopic ACTH production was present in the cytoplasm of both cell types, irrespective of the absence (Figure 2D) or presence of mutations (Figure 2E) in the *ARMC5* gene. Next, we investigated whether there was a difference in the steroidogenic function of spongiocytes and compact cells by analyzing StAR protein and the CYP17A1 and 3BHSD2 enzymes. For comparison, the distribution of these proteins and enzymes was analyzed in normal adrenal tissue, showing a homogenous pattern of distribution (Figure S2C-S2J). We observed a heterogeneous distribution of StAR in the cytoplasm of both compact cells and spongiocytes (Figure 3A and 3B), and no differences were found in the percentages of the StAR-positive area in nonmutated and mutated patient sections (Figure 3C). The 3BHSD2 enzyme was distributed in the cytoplasm of spongiocytes in nonmutated and mutated patient sections but not in compact cells (Figures 3D and 3E). Moreover, patients carrying *ARMC5* mutations showed more 3BHSD2-positive cells than those not carrying mutations (p = 0.016), with percentages of 36.7 2.8% and 31.1 ± 4.1%, respectively (Figure 3F). However, the relative *3BHSD2* gene expression was not different between the spongiocytes of mutated and nonmutated patients (Figure 3G). The CYP17A1 enzyme was present in both cell types but was more intensely expressed in the compact cells than in spongiocytes (Figures S3A and S3B). The investigation of relative *CYP17A1* gene expression showed no differences between the spongiocytes of mutated and nonmutated cells (Figure S3C). We next investigated the presence of the most frequently studied aberrant receptors described in PMAH (3,16,17). When analyzing the vasopressin receptor AVP1Ra, we observed a homogeneous pattern of cytoplasmic expression in both spongiocytes and compact cells irrespective of the presence or absence of ARMC5 mutation (Figure 4A-C); this result was in agreement with the microdissection analysis of gene expression in spongiocytes (Figure 4D). The presence of the serotonin receptor 5HT4R was observed in both cell types (Figures 4E and 4F). Moreover, the nodules of patients carrying *ARMC5* mutations presented with more 5HT4R-positive cells than those who did not carry *ARMC5* mutations (Figure 4G). For comparison, the presence of 5HT4R in normal human adrenal tissue was analyzed, and 5HT4R labeling was not observed in the normal adrenal cortex (Figures S2I and S2J). However, the microdissection analyses revealed similar *5HT4R* gene expression between the mutated and nonmutated patient groups (Figure 4H). Finally, we did not observe the presence of GIPR protein in the sections of the macronodules studied here (Figures S4A and S4B) despite the presence of mRNA in the microdissection analyses of spongiocytes from mutated and nonmutated tissue sections (Figure S4C).

## DISCUSSION

In this study, we investigated the composition and functional patterns of PMAH nodules and whether mutations in the *ARMC5* gene could interfere with them. First, we observed the presence of the ARMC5 protein in the cell cytoplasm in both cell types of nonmutated and mutated patient sections, as done by Assié and cols. (7), and in cell cultures obtained from PMAH nodules (14). Through microdissection analysis, we observed that the *ARMC5 *mRNA expression in spongiocytes was higher in mutated patients than in nonmutated patients, while we observed a heterogeneous expression of ARMC5 in PMAH cell cultures, with a trend toward a lower expression of ARMC5 in mutated cells than in nonmutated cells (14). The consequences of the mutations in *ARMC5* on transcription have not yet been elucidated; therefore, we can only speculate that distinct somatic mutations would have different consequences on ARMC5 expression; that is, some somatic mutations would be more deleterious than others. However, this remains to be established. Additionally, it is important to emphasize that the results of this study were obtained from spongiocyte microdissected tissue, while the results from the previous study of our group were obtained from cell cultures with both cell types, spongiocytes and compact cells, which could explain the difference between the results. However, this remains to be confirmed.

*ARMC5* may be a tumor suppressor gene, but its function is still unclear. An analysis of the expression of the four *ARMC5* isoforms in 46 normal human tissues showed that at least one was ubiquitously expressed throughout the body, whereas the adrenal gland expressed all isoforms (13). The extensive expression of *ARMC5* in different tissues suggests additional physiological functions as well as *ARMC5* involvement in the pathologies of other tissues.

Moreover, our results from the PCNA protein analysis suggest a higher proliferative capacity of nodules carrying mutations in the *ARMC5* gene than that of those without *ARMC5* mutations. Since *ARMC5 *is considered a putative tumor suppressor gene and because the origin of macronodules in PMAH is not known, mutations in *ARMC5* might influence proliferation or apoptosis (7,14), causing adrenal growth. However, the possibility of unknown variables cannot be excluded.

The macronodules analyzed in this study presented the same characteristics as those described in a study by Sasano and cols. (2), in which differential distribution of the CYP17A1 and 3βHSD2 enzymes in spongiocytes and compact cell types was observed, but a homogeneous distribution of the StAR protein was observed. It seems that mutations in *ARMC5 *did not interfere with the quantity of steroidogenic enzymes in our cohort, which is in contrast with the quantity of the CYP11A1 protein described in a mutated patient by Assié and cols. (7). We hypothesize that other unknown factors may lead to this difference since PMAH is a very heterogeneous disease and each patient could present different secondary factors.

To date, there is no known direct relationship between ARMC5 mutations and the presence of aberrant receptors in PMAH. However, we interestingly observed a higher number of 5HT4R-positive cells in the nodules of patients carrying *ARMC5 *mutations than in the nodules of patients without *ARMC5* mutations. Bertherat and cols. (18) suggested that the presence of 5HT receptors might include a serotonergic regulation of cortisol secretion. Moreover, Louiset and cols. and Le Mestre and cols. reported the stimulation of adrenal steroidogenesis by serotonin receptors in the presence of specific 5-HT agonists (6,19). We hypothesize that the increased presence of 5HT4 receptors would increase cortisol production in patients carrying mutations in *ARMC5,* and this would contribute to a more severe clinical presentation.

Swords and cols. (20) reported a case in which aberrant expression of *GIPR* mRNA was not sufficient to cause food-dependent CS. Here, we observed heterogeneous mRNA expression of *GIPR* among the investigated patients without detection of the GIPR protein, suggesting that the mRNA expression in these patients was not sufficient to produce detectable amounts of protein.

Finally, our findings suggest that *ARMC5*-mutated PMAH nodules are related to higher proliferation rates and to a greater presence of the 5HT4R than nonmutated nodules. These new data increase the understanding of the origin of macronodules and could help explain the clinical presentation in this subset of ARMC5-mutated patients.
